# Pregestational Body Mass Index Is Associated with Differential Expression of ABC Transporter in the Maternal–Neonatal Dyad

**DOI:** 10.3390/metabo16100734

**Published:** 2026-09-30

**Authors:** America Vanoye-Carlo, Sandra Berenice Parra-Hernandez, Ameyalli M. Rodríguez-Cano, Otilia Perichart-Perera, Mari Cruz Tolentino-Dolores, Marco Cerbón, Oscar A. Perez-González, Sonia Nava-Salazar

**Affiliations:** 1 Laboratorio de Oncología Experimental, Instituto Nacional de Pediatría, SSA., Mexico City 04530, Mexicooperezg@pediatria.gob.mx (O.A.P.-G.); 2 Departamento de Inmunobioquímica, Instituto Nacional de Perinatología Isidro Espinosa de los Reyes, Mexico City 11000, Mexico; rebe1602@hotmail.com; 3 Coordinación de Nutrición y Bioprogramación, Instituto Nacional de Perinatología Isidro Espinosa de los Reyes, Mexico City 11000, Mexico; ameyalli.rodriguez@inper.edu.mx (A.M.R.-C.); otiliaperichart@inper.gob.mx (O.P.-P.); cruz_tolentino@yahoo.com.mx (M.C.T.-D.); 4 Facultad de Química, Universidad Nacional Autónoma de México, Mexico City 04510, Mexico; marcocerbon@quimica.unam.mx

**Keywords:** ABC transporters, pregestational BMI, pregnancy, obesity, newborn

## Abstract

**Background**: Maternal obesity has been associated with fetal growth abnormalities and alterations in metabolic programming. ATP-binding cassette transporters, including ABCA1, ABCG1, ABCC1 and ABCG2, play essential roles in cholesterol homeostasis and placental function, fetal growth and development. However, the regulation of these transporters in the maternal–fetal axis under metabolic stress remains poorly understood. **Methods**: We evaluated 92 mother–newborn pairs, stratified by pregestational body mass index into three groups: normal weight (*n* = 34), overweight (*n* = 35) and obese (*n* = 23). Metabolic profiles and *ABCA1*, *ABCG1*, *ABCC1* and *ABCG2* mRNA expression in peripheral blood mononuclear cells were assessed. **Results**: Obese mothers demonstrated significantly higher TyG-BMI indices compared to normal-weight women. Regarding transporter expression, *ABCA1* levels were elevated, while *ABCG1* and *ABCG2* were significantly downregulated in both overweight and obese mothers and their newborns. Positive correlations were observed between maternal and neonatal *ABCA1*. Maternal metabolic status correlated with ABC transporter expression, as demonstrated by inverse relationships between fasting glucose and maternal *ABCA1* expression and between cholesterol levels and maternal *ABCG2* expression. Furthermore, *ABCG1* and *ABCG2* expression levels positively correlated with fetal growth parameters. **Conclusions**: Maternal overweight and obesity before pregnancy were associated with significant changes in the expression of key ABC transporters in both mothers and their newborns. Changes in maternal *ABCA1* and *ABCG1* expression could be related to altered cholesterol homeostasis, while reduced *ABCG2* expression possibly reflects changes in pathways involved in cellular protection and the handling of potentially harmful compounds. Further longitudinal studies are required to clarify underlying mechanisms and determine their persistence postnatally.

## 1. Introduction

During pregnancy, the body experiences significant physiological changes, particularly due to the formation of the placenta. The placenta must balance two competing demands: transporting nutrients and endocrine signals to the fetus while simultaneously blocking xenobiotics, oxidized lipids and harmful maternal metabolites [1,2].

Cholesterol plays a crucial role in fetal development, as it is essential for the formation of cell membranes and various signaling functions. To meet the demands of maternal and placental steroid production, elevated maternal cholesterol levels are necessary. Several factors influence lipid levels during pregnancy and the extent of these changes, including pre-pregnancy lipid levels, body mass index (BMI), age, diet and ethnicity [3,4].

Maternal lipoproteins bind to receptors in the placenta, which facilitates the transport of cholesterol and triglycerides into the fetal circulation. Fetal cholesterol comes from two sources: de novo synthesis and maternal circulation. Research has shown that cholesterol precursor levels in the amniotic fluid significantly increase in the second trimester, indicating a growing reliance on maternal cholesterol, particularly as fetal cholesterol levels correlate with maternal plasma levels. Importantly, plant sterols in newborns, derived from the maternal diet, also successfully transfer through the placenta [5,6].

Maternal metabolic dysfunction is linked to abnormalities in fetal growth and body composition, which can be attributed to changes in placental barrier protection, nutrient transfer and detoxification processes. These alterations can lead to adverse perinatal outcomes and an increased risk of metabolic disorders later in life. One notable consequence is increased neonatal adiposity, characterized by excess lipid accumulation and elevated glucose levels, as well as higher maternal cholesterol and triglyceride levels [1,7,8].

ABC transporters constitute a mechanism of cholesterol homeostasis during gestation; specifically, P-glycoprotein (P-gp/ABCB1) and breast cancer resistance protein (BCRP/ABCG2) are primarily expressed in the apical brush-border membrane of the syncytiotrophoblast. This expression helps prevent maternal compounds from passing into fetal circulation. Additionally, transporters from the ABCA and ABCC subfamilies play critical roles in maintaining placental sterol balance, bile acid transport, steroid hormone metabolism and immunological processes. Notably, the regulation of these transporters is dynamic and influenced by various hormonal and other factors, making them susceptible to disruption [9,10].

ABCG1, which is primarily expressed in macrophages, exports cholesterol to high-density lipoprotein (HDL) and facilitates reverse cholesterol transport by sending cholesterol from the peripheral tissues back to the liver or gut for excretion [11,12]. ABCA1 contributes to HDL cholesterol formation and reverse cholesterol transport [13], while ABCC1 and ABCG2 are mainly involved in drug transport, with their activity affected by cholesterol levels. ABCG2, located in cholesterol-rich membranes, exhibits reduced functionality when cholesterol content is low. Similarly, decreased cholesterol levels cause ABCC1 to relocate within the membrane, impacting its performance [14,15,16].

Given the importance of ABC transporters in fetal nutrition, any disruption in their regulation could lead to an imbalance in metabolic processes. This study investigates whether the expression of ABC genes—ABCA1, ABCC1, ABCG1 and ABCG2—varies in mother–newborn pairs in relation to maternal factors, such as BMI during pregnancy, as well as how this relates to neonatal adiposity and metabolic profiles.

## 2. Materials and Methods

### 2.1. Study Population

This study was approved by the Institutional Review Board of the Instituto Nacional de Perinatología in Mexico City (212250-3210-21002-05-16). Participants were consecutively recruited for this prospective study between June 2015 and March 2020. The surgical schedule was reviewed regularly, and medical records were screened to identify women meeting the eligibility criteria. Eligible participants were invited to enroll upon hospital admission for scheduled cesarean delivery. The study protocol was explained in detail and written informed consent was obtained from all participants before enrollment.

The sample size calculation was based on the three study groups: women with normal weight, overweight, and obesity. In Mexico, combined reported prevalence of overweight and obesity is 70%. The minimum required sample size was calculated using an online statistical calculator (Department of Statistics, University of British Columbia; http://www.stat.ubc.ca/~rollin/stats/ssize/b2.html, accessed on 31 January 2015) by comparing independent samples on a two-sided test, assuming a significance level of 0.05 and 80% statistical power. This yielded a minimum sample size of 24 cases per BMI category (totaling 72 participants). A total of 92 mothers were ultimately included in the study after applying the predefined eligibility criteria.

Healthy women with full-term pregnancies undergoing scheduled cesarean section with no evidence of labor were included. Inclusion criteria were ≥18 years old, no comorbidities, no treatment affecting carbohydrate or lipid metabolism, singleton pregnancies and a fetus without structural congenital malformation.

Demographic and medical data were collected from medical records, including participants’ age, pregestational weight (measured before the 12th week of gestation), height, gravidity, parity, diastolic blood pressure and systolic blood pressure. A pregestational Body Mass Index (BMI) was calculated as weight in kilograms divided by the square of the height in meters. It was then categorized as follows: normal weight (18.5 ≤ BMI < 25.0 kg/m^2^), overweight (25.0 ≤ BMI < 30.0 kg/m^2^) or obese (BMI ≥ 30.0 kg/m^2^) based on the World Health Organization definitions. Gestational weight gain (GWG) was calculated based on the Institute of Medicine (IOM) guidelines [17].

### 2.2. Sample Collection

Maternal blood samples were collected during fasting and those of the newborns at the time of cesarean section. Blood serum was separated and immediately frozen at −80 °C. Peripheral Blood Mononuclear Cells (PBMCs) were isolated from 5 mL of blood in a Lymphoprep density gradient (StemCell Technologies, Vancouver, BC, Canada). Samples were processed and immediately frozen until use.

### 2.3. Laboratory Measurements

Serum biochemical parameters were measured using commercial kits according to the manufacturer’s protocols on EchoPlus 2000 biochemistry analyzer (Rome, Italy), which included total cholesterol (TC), triglycerides (TG), high-density lipoprotein cholesterol (HDL-C) and low-density lipoprotein cholesterol (LDL-C). The calculation formula for the TyG index is as follows: TyG index = Ln [fasting triglycerides (mg/dL) × fasting blood glucose (mg/dL)/2] [18]. The cut-off value was greater than 9.27, based on the study by Wang et al. [19], which involved women in their third trimester and Adequate for Gestational Age newborns. The TyG-BMI is defined as Ln [fasting triglycerides (mg/dL) × fasting blood glucose (mg/dL)/2] * BMI. A cut-off value greater than 211.03 was derived from a first-trimester study, as no references exist for full-term pregnancy [20]. TyG ≥ 7.8 is recommended as a cut-off point in neonates [21,22].

### 2.4. Anthropometric Assessment

Measurements were taken on newborns during the first 72 h after birth. These included weight, length and head circumference. Using this data, we calculated the Fetal Medicine Foundation (FMF) percentile, Fetal Medicine Barcelona (FMB) percentile, Intergrowth percentile, FMF z score and Intergrowth z score. These percentiles help classify newborns into three categories: Small for Gestational Age (SGA, below the 10th percentile); Adequate for Gestational Age (AGA, between the 10th and 90th percentiles); and Large for Gestational Age (LGA, above the 90th percentile). This classification allows the growth status of the newborns to be assessed effectively.

### 2.5. Real-Time Quantitative PCR of mRNA Levels

Total RNA was isolated from PBMCs using the RNeasy Mini Kit (Qiagen, Hilden, Germany, 74104). cDNA was synthesized using SuperScript III First-Strand Synthesis Super Mix (Invitrogen, Carlsbad, CA, USA, 18080–400), according to the manufacturer’s instructions. Real-time RT-qPCR was performed using a StepOnePlus^TM^ Real-Time PCR System (Applied Biosystems, Foster City, CA, USA). The TaqMan probes used were: ABCA1, FAM Hs01059118_m1; ABCG1, Hs00245154_m1; ABCC1, Hs01561502_m1; ABCG2, FAM Hs01053790_m1 and eukaryotic 18S rRNA (4319413E) as the endogenous control (Applied Biosystems, Foster City, CA, USA). Results were taken as cycles of threshold (CTs). All TaqMan probes demonstrated efficiency within the acceptable range of 90 to 110%.

RNA integrity was assessed before cDNA synthesis. Only samples with an RNA Integrity Number (RIN) greater than 8 and acceptable spectrophotometric purity (A260/A280 ratio) were included in the analyses. The amplification protocol, following the manufacturer’s recommendations, consisted of a 50 °C holding step for 2 min, a 95 °C polymerase activation step for 10 min, 40 cycles of 95 °C for 15 s and 60 °C for 1 min. Each sample underwent technical triplicate analysis and reactions exhibiting a cycle threshold (Ct) difference exceeding 0.5 cycles among replicates were repeated. Only reactions that satisfied this quality criterion were included in the final analysis. Negative controls, including no-template controls, were incorporated in every qPCR run to monitor potential contamination, with no amplification detected in these controls. To minimize analytical variability or potential batch effects, all qPCR reactions were conducted using the same instrument and reagent, as well as primer batches, cycling conditions and a standard protocol. Furthermore, samples from different study groups were distributed across qPCR runs and analyzed under identical experimental conditions whenever feasible. Relative gene expression was calculated using the 2^−ΔΔCT^ method [23] and normalized to the endogenous control 18S rRNA.

### 2.6. Statistical Analysis

Continuous variables were presented as mean ± standard deviation (SD), while categorical variables were summarized as frequencies and percentages. Depending on the data distribution, differences among groups were analyzed using one-way analysis of variance (ANOVA) or the Kruskal–Wallis test. When significant differences were detected, multiple pairwise comparisons were performed using Tukey’s post hoc test for parametric data or Bonferroni-adjusted pairwise comparisons for non-parametric data. Categorical variables were compared using Pearson’s chi-square test. Spearman’s correlation coefficient was used to assess associations among biochemical parameters, fetal growth parameters and transporter expression levels. Confounding factors, including age, parity, tobacco use before pregnancy, and the sex of the newborn, were analyzed. Statistical significance was defined as *p* ≤ 0.05. All analyses were conducted using IBM SPSS Statistics software version 25.0 (IBM Corporation, Armonk, NY, USA).

## 3. Results

Approximately 105 pregnant women were initially enrolled using a consecutive selection process. Participants were excluded due to insufficient biological sample volume or technical issues encountered during sample collection, processing or laboratory analyses. The final study population comprised 92 pregnant women: 34 with a normal pre-pregnancy BMI, 35 classified as overweight and 23 classified as obese. Newborn samples were also excluded due to collection difficulties, insufficient sample volume or technical problems during laboratory analyses. All included participants underwent metabolic profiling and gestational weight gain (GWG) was assessed. Table 1 summarizes demographic and biochemical parameters of pregnant women by BMI category.

Women in the obese group were younger on average compared to those in the normal weight and overweight groups. GWG decreased as pregestational BMI increased and women with a normal BMI category gained the most weight during pregnancy, according to previous reports and current gestational weight gain recommendations [24,25].

When categorizing GWG according to IOM guidelines, the highest percentage of women with insufficient weight gain (IWG) was observed in the normal BMI group (41.2%). In comparison, excessive weight gain (EWG) was more frequent among overweight women (37.1%) and adequate weight gain (AWG) was most common in the obese group (69.6%). The proportion of primigravid women was similar in the normal weight and obese groups, at 21.4% and 20% respectively, but was lower in the overweight group at 10.5%. There was no significant difference in smoking status among the normal-weight, overweight and obese groups (38.2%, 25.7% and 39.1%, respectively).

Regarding metabolic markers, fasting glucose levels were slightly higher in the obese group (81.21 ± 12.67 mg/dL), though no significant differences were noted across groups. Total cholesterol and triglyceride levels tended to be higher in the overweight and obese groups compared to the normal weight group. HDL-C levels were highest in the normal BMI group (65.35 ± 10.47 mg/dL), while LDL-C levels increased progressively with BMI, reaching the highest values in the obese group (150.75 ± 30.12 mg/dL). The TyG-BMI was statistically significantly higher in the group of obese mothers (298.82 ± 35.66) compared to the overweight (248.65 ± 17.80) and both groups are above the reference cut-off point. The normal weight group was below the cut-off point established for this index (198.11 ± 16.29). There were no statistically significant differences among the groups in the TyG index and the scores for the three study groups were below the cut-off point. Table 2 summarizes the biochemical and anthropometric parameters of newborns, categorized by their mothers’ pregestational BMI. Twenty-five newborns of women with normal BMI, 27 of overweight women and 20 of obese women were included.

Gestational age at birth was slightly lower in the overweight (38.63 ± 1.28) and obese groups (38.85 ± 1.66) compared to the normal weight group (39.1 ± 1.07), though differences were not statistically significant. Newborn weight was highest among offspring of overweight mothers (3248.42 ± 520.52 g), followed by those from the obese (3114.23 ± 550.97 g) and normal weight groups (3033.62 ± 373.83 g). In terms of biochemical parameters, fasting glucose levels in newborns were slightly higher in the obese group (67.50 ± 6.68 mg/dL). In contrast, the TyG index, total cholesterol, triglycerides, HDL-C and LDL-C levels were comparable across all groups, with no significant trends observed in relation to maternal BMI. None of these parameters showed statistically significant differences among the groups.

Fetal growth assessed using the FMF and FMB scales as well as Intergrowth. Fetal percentiles showed a tendency to be lower in the obese group, whereas the Intergrowth percentile was notably higher in the obese group (66.35 ± 29.81), with a corresponding positive z-score (0.64 ± 1.13), compared to negative z-scores in the normal and overweight groups. The distribution of newborns by size for gestational age revealed that the highest proportion of SGA infants was observed in the obese group (30%). In comparison, the overweight group had the highest proportion of LGA infants (22.22%).

We evaluated mRNA expression of maternal and newborn transporters *ABCA1*, *ABCG1*, *ABCC1* and *ABCG2*. The mRNA expression profiles of these ABC transporters in mothers and newborns are presented in Figure 1 and Figure 2. In maternal samples, *ABCA1* mRNA expression was significantly increased in both overweight and obese groups compared to the normal-weight controls. Conversely, *ABCG1* and *ABCG2* mRNA levels were significantly reduced in obese women compared to normal and overweight mothers. No significant differences were observed in *ABCC1* mRNA expression across the maternal groups.

A similar pattern was observed in newborns of overweight and obese mothers. They showed a significant increase in *ABCA1*, and *ABCC1* mRNA expression compared to those born to normal weight mothers. On the other hand, *ABCG1* and *ABCG2* mRNA expression levels were significantly reduced in newborns of both overweight and obese mothers. Overall, maternal overweight and obesity were associated with marked dysregulation of ABC transporter expression in both the mother and newborns, characterized primarily by increased *ABCA1* mRNA expression but reduced *ABCG1* and *ABCG2* mRNA expression.

Correlation analyses were performed between transporter expression levels and the different parameters of the mother and newborn. Table 3 presents Spearman correlation coefficients for associations involving maternal and neonatal ABC transporters: *ABCA1*, *ABCG1*, *ABCC1* and *ABCG2*.

Moderate, positive correlations were observed between maternal *ABCA1* and maternal *ABCG1* (r = 0.447, *p* = 0.0001), as well as between maternal *ABCA1* and neonatal *ABCA1* (r = 0.623, *p* = 0.017). Additionally, moderate, positive correlations were found between the expression of neonatal transporters *ABCA1* and *ABCC1* (r = 0.558, *p* = 0.0001). In contrast, a moderate negative correlation was identified between the neonatal *ABCA1* and *ABCG1* (r = −0.526, *p* = 0.0001), as well as between neonatal *ABCA1* and *ABCG2* (r = −0.478, *p* = 0.001). Neonatal *ABCG1* mRNA expression was moderately negatively correlated with neonatal *ABCC1* expression (r = −0.546, *p* = 0.0001). The results also indicated a moderate negative correlation between neonatal *ABCC1* and *ABCG2* (r = −0.663, *p* = 0.001). Furthermore, a single strong, positive correlation was observed between the expression of neonatal *ABCG1* and *ABCG2* (r = 0.748, *p* = 0.0001).

After adjustment for potential confounders such as maternal age, primigravida status, pre-pregnancy smoking and neonatal sex, only the positive association between maternal *ABCA1* and maternal *ABCG1* mRNA expression and between maternal and neonatal *ABCA1* mRNA expression remained statistically significant (Table S1, Figures S1 and S2).

In addition, mRNA expression of maternal and fetal transporters correlated with the biochemical and fetal growth indicators in newborns; the Spearman correlation coefficients are presented in Table 4. Maternal fasting glucose demonstrated a moderate negative correlation with maternal *ABCA1* mRNA expression (r = –0.508, *p* = 0.037) (Figure S2A). Similarly, maternal total cholesterol was negatively correlated with maternal *ABCG2* mRNA expression (r = –0.515, *p* = 0.034, Figure S2B). These associations remained significant after adjustment for confounding variables (Table S2). Following adjustment for potential confounding variables, maternal *ABCG1* and *ABCG2* mRNA expression levels were positively correlated with FMF percentile (r = 0.569, *p* = 0.001; r = 0.418, *p* = 0.001, respectively) (Figure S2C,D). A moderate to strong correlation was initially observed between mRNA levels of *ABCG1*, *ABCC1* and *ABCG2* in newborns and the TyG-BMI; however, these associations did not remain significant after adjustment for confounding variables (Table S3).

## 4. Discussion

In the present study, we evaluated the effects of pregestational BMI and maternal metabolic status on the expression of ABC family transporters in mothers and their newborns. The findings suggest that maternal adiposity before pregnancy could be associated not only with metabolic alterations in the mother, but also with changes in the expression profile of lipid- and xenobiotic-related transporters in both maternal and neonatal compartments. These results support the concept that maternal metabolic status could influence fetal physiology through regulatory mechanisms involving ABC transporters.

According to IOM guidelines, women with a normal pregestational BMI may gain more weight than those who are overweight or obese. Interestingly, inadequate GWG was more frequent among women with normal BMI, whereas excessive GWG predominated in overweight women. Similar findings have been previously described and may reflect the implementation of stricter nutritional counseling and medical surveillance in women with obesity during pregnancy [24,26]. These observations highlight the importance of individualized GWG recommendations and monitoring strategies based on maternal BMI and metabolic profile, rather than relying exclusively on generalized guidelines [27].

Analysis of blood serum lipid levels showed no significant differences between the groups. However, obese women showed a non-significant trend toward higher LDL cholesterol and lower HDL cholesterol, trends that could point to early stages of metabolic dysregulation. The TyG-BMI exceeded the established cut-off value in overweight and obese women, supporting this idea.

The TyG-BMI is a useful measure of insulin resistance and is linked to a higher risk of heart disease, metabolic issues, and cancer [28,29]. In overweight and obese pregnant women, the observed metabolic alterations could be early signs of ongoing metabolic problems that make them susceptible to developing metabolic syndrome, type 2 diabetes, and heart disease later in life [30].

Maternal obesity can affect fetal growth, increasing the chances of having neonates who are either LGA or SGA. In our study, we did not find significant differences in birth weights among the mothers we observed. This could be due to our small sample size, which limits our ability to draw strong conclusions. Additionally, maternal obesity leads to changes in the placenta. These changes affect how the placenta transports fats, processes sugar, signals inflammation, and senses nutrients. These alterations could influence how much nutrition the fetus receives and how it grows, even when clear outcomes like macrosomia were not observed. The absence of differences in birth-weight categories does not necessarily exclude the presence of obesity-related placental and metabolic alterations that could affect fetal development [31,32].

We evaluated fetal growth using the FMF, FMB, and Intergrowth scales and found different patterns based on mothers’ BMI categories. Previous studies showed that FMB and Intergrowth provide the best predictive performance for fetal growth assessment in the Mexican population [33]. In our study, newborn percentiles in the normal BMI group were very similar between these two scales. In the overweight group, all three scales gave similar results. However, in the obese group, the FMF and FMB scales showed more agreement. These differences could come from the various clinical and biometric factors included in each growth standard. This highlights the importance of using growth references that fit the specific population. Despite the differences in scale values, all newborns demonstrated normal growth, regardless of their mothers’ pregestational BMI.

One of the most relevant findings of this study was the differential expression of ABC transporters according to maternal BMI status. ABCA1 mRNA expression was significantly upregulated in both mothers and newborns, whereas ABCG1 mRNA expression was significantly downregulated in women with obesity and in newborns of overweight and obese women, compared to those of normal weight.

Because of the complementary roles of ABCA1 and ABCG1 in cellular cholesterol efflux, these results could indicate differences in the regulation of cholesterol transport related to maternal BMI. ABCA1 is responsible for initiating the efflux of cholesterol to lipid-poor apoA-I, which aids in the formation of nascent HDL. In contrast, ABCG1 facilitates the transfer of cholesterol to mature HDL particles [13,34]. A reduction in ABCG1 mRNA expression could be associated with inefficient cholesterol clearance; however, this hypothesis should be explored in future studies that examine ABC transporter regulation at various levels.

Similarly, *ABCG2* mRNA expression was markedly decreased in mothers with obesity and their newborns. ABCG2 is a key placental ATP-binding cassette transporter that functions as a major efflux system for xenobiotics, environmental contaminants and endogenous metabolites, thereby contributing to fetal protection during pregnancy [35,36]. In vitro experimental studies have shown that environmental toxicants, including pesticides and heavy metals such as cadmium, can downregulate placental *ABCG2* mRNA expression and protein, as well as activity, potentially impairing the placental barrier against xenobiotic exposure [37,38,39]. In our cohort, the decrease in *ABCG2* mRNA expression associated with maternal obesity could be linked to changes in transporter regulation due to obesity-related metabolic alterations. However, to fully understand the protein’s regulation and activity and its relationship to cholesterol, it is essential to investigate both protein expression and post-translational modifications of the transporter.

ABCC1 has been linked to the cellular export of glucocorticoids, playing a role in the tissue-specific modulation of glucocorticoid actions. Previous studies reported that ABCC1 preferentially transports corticosterone and could affect the availability of glucocorticoids within tissues like adipose tissue [40,41,42]. The regulation of the ABCC1 transporter in newborns, particularly in the context of maternal obesity, is important, as it possibly helps limit glucocorticoid effects related to insulin resistance and glucose regulation. However, the current data do not allow us to determine whether the increased *ABCC1* mRNA expression observed in the newborns of overweight and obese women leads to changes in transporter activity or glucocorticoid exposure.

To explore the relationship between transporter expression in newborns and their mothers, a Pearson correlation analysis was conducted, adjusting for confounding factors. We also examined maternal obesity-related factors and newborn growth and biochemical parameters in relation to transporter expression. The results indicate a possible correlation between maternal and neonatal transporter expression, suggesting a coordinated maternal–fetal relationship in cholesterol homeostasis regulation.

One significant finding was the association between maternal and neonatal *ABCA1* mRNA expression, which could be linked to metabolic changes in the mother. ABCA1 is known to play a crucial role in regulating the efflux of cholesterol from cells and maintaining lipid balance. This function is particularly relevant in the context of maternal–fetal interactions, as it could influence cholesterol transport during pregnancy. The observed relationship between neonatal and maternal *ABCA1* expression suggests that both maternal and fetal factors share common regulatory mechanisms. Future research on the regulation of the ABCA1 process during pregnancy is essential to better understand the interplay between maternal and fetal cholesterol regulation.

Maternal expression levels of *ABCG1* and *ABCG2* mRNA were associated with the FMF percentile, an established indicator of fetal growth. This association suggests that these transporters could play a role in fetal growth and development. Previous studies have reported reduced placental *ABCG2* mRNA expression in pregnancies complicated by intrauterine growth restriction, supporting the hypothesis that ABCG2 contributes to placental viability and the regulation of fetal growth [43]. Although the role of ABCG1 in fetal growth is less well defined, the present findings suggest that both transporters could participate in pathways involved in fetal maturation and nutrient exchange.

A negative correlation between maternal fasting glucose and *ABCA1* mRNA expression has been reported in earlier studies. Existing data indicate that hyperglycemia can suppress *ABCA1* mRNA expression independently of classical transcriptional regulators, which suggests the involvement of alternative regulatory mechanisms [44].

The negative association found between maternal cholesterol levels and maternal *ABCG2* mRNA expression contrasts with previous evidence suggesting a positive relationship between cholesterol availability and *ABCG2* expression and/or activity [45,46]. Cholesterol is known to be important for the optimal activity of the ABCG2 transporter, indicating that the relationship between cholesterol homeostasis and ABCG2 could involve both transcriptional and post-translational mechanisms. It is essential to interpret the inverse association observed in our study with caution. This discrepancy could be due to variations in cellular or physiological contexts, tissue-specific regulatory mechanisms, changes in cholesterol-derived signaling molecules, or other metabolic factors linked to maternal cholesterol status. Additionally, since our analysis focused on *ABCG2* mRNA expression in maternal PBMC, further studies on protein expression and function are necessary to better understand the regulation of ABCG2 during pregnancy and its role in fetal development.

Taken together, our findings support the hypothesis that maternal and neonatal ABC transporters are coordinated and associated with maternal metabolic status, fetal growth and developmental maturation. The expression profiles observed in overweight and obese women suggest the presence of adaptive, yet potentially maladaptive, mechanisms affecting cholesterol transport and cellular defense pathways under conditions of maternal metabolic stress.

The results of this study suggest a close regulation of ABC transporter expression in both mothers and newborns, highlighting the significance of the mother’s metabolic status. However, further detailed research into these interactions is essential. Our study was limited by a small number of participants and reported events of interest, which restricted our ability to conduct a more comprehensive analysis that accounts for factors such as lifestyle, socioeconomic status, dietary habits, and environmental influences. It is important to note that measurements were taken from blood samples, as obtaining PBMCs is a minimally invasive procedure that offers insights into systemic status and is commonly used to assess maternal and neonatal metabolic health. Analyzing the expression and function of the involved proteins as well as detailed studies using “in vitro” and animal models could help to elucidate the underlying cellular mechanisms of ABC transporters coregulation involved in the maternal–fetal dyad. Furthermore, given our findings, it is vital to implement long-term monitoring of offspring development. This proactive approach can help clarify the connections between transporter expression and function, particularly in relation to metabolic and cardiovascular outcomes.

## 5. Conclusions

In conclusion, being overweight or obese before pregnancy is linked to subtle metabolic changes and significant alterations in the expression of key ABC transporters in both mothers and their newborns. Among mothers, an imbalance in *ABCA1* and *ABCG1* mRNA expression, along with lower *ABCG2* mRNA expression, was observed by pregestational BMI category.

The observed differences in newborns could be related to molecular adaptations to the intrauterine metabolic environment. Associations between maternal and neonatal transporter expression suggest a close maternal–fetal regulatory relationship. Additional longitudinal and functional research, including ethically appropriate follow-up of newborns, is required to clarify the mechanisms underlying these associations and to assess whether the observed molecular patterns persist throughout postnatal development.

## Figures and Tables

**Figure 1:**
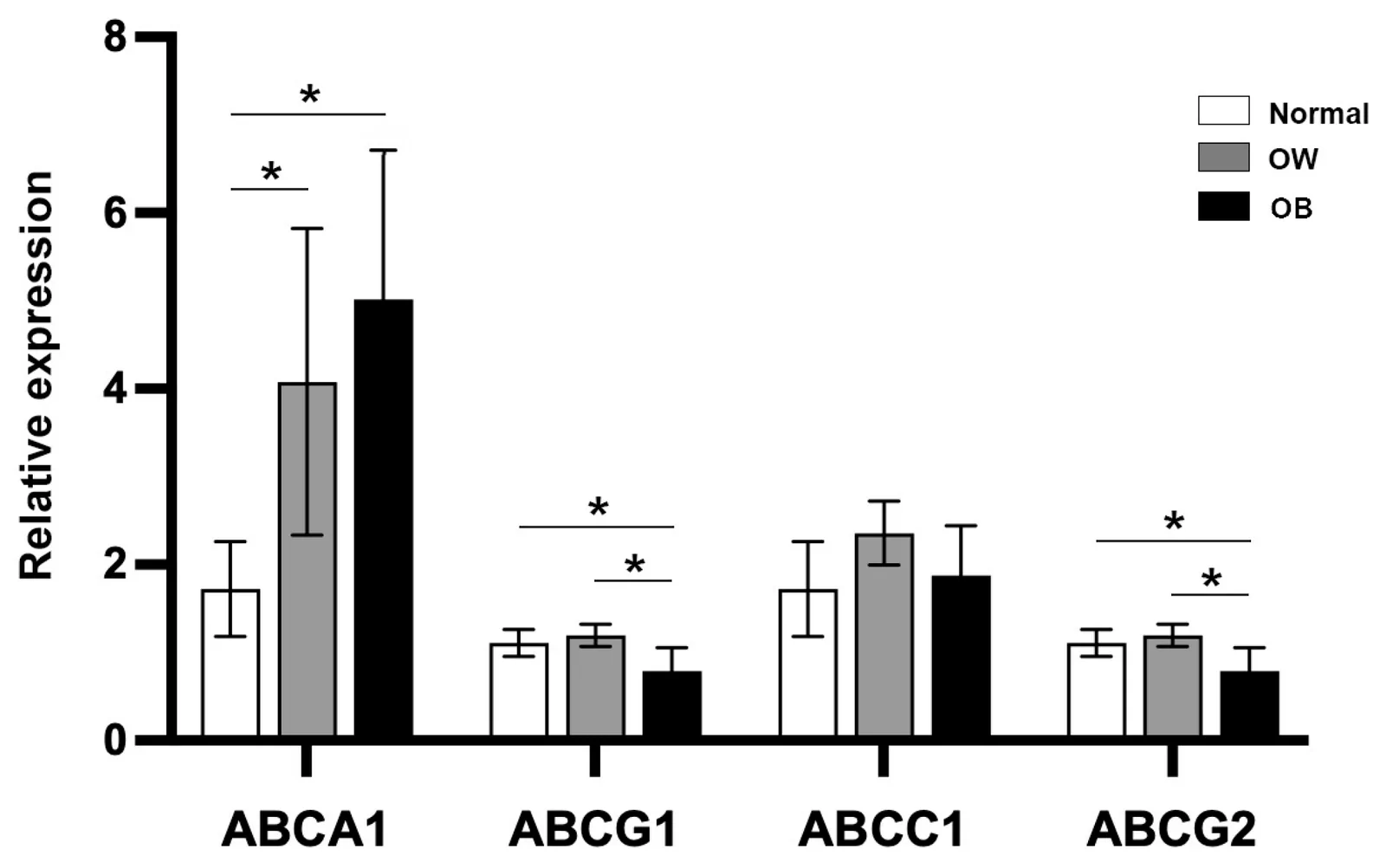
Maternal expression of ABC transporters. Gene expression analysis of *ABCA1*, *ABCG1*, *ABCC1* and *ABCG2* in women categorized by pregestational weight: normal (Normal, white), overweight (OW, gray) and obesity (OB, black). Statistical significance was assessed using the Kruskal–Wallis test, followed by Bonferroni-adjusted pairwise comparisons. Data are presented as mean ± SD. * *p* < 0.05.

**Figure 2:**
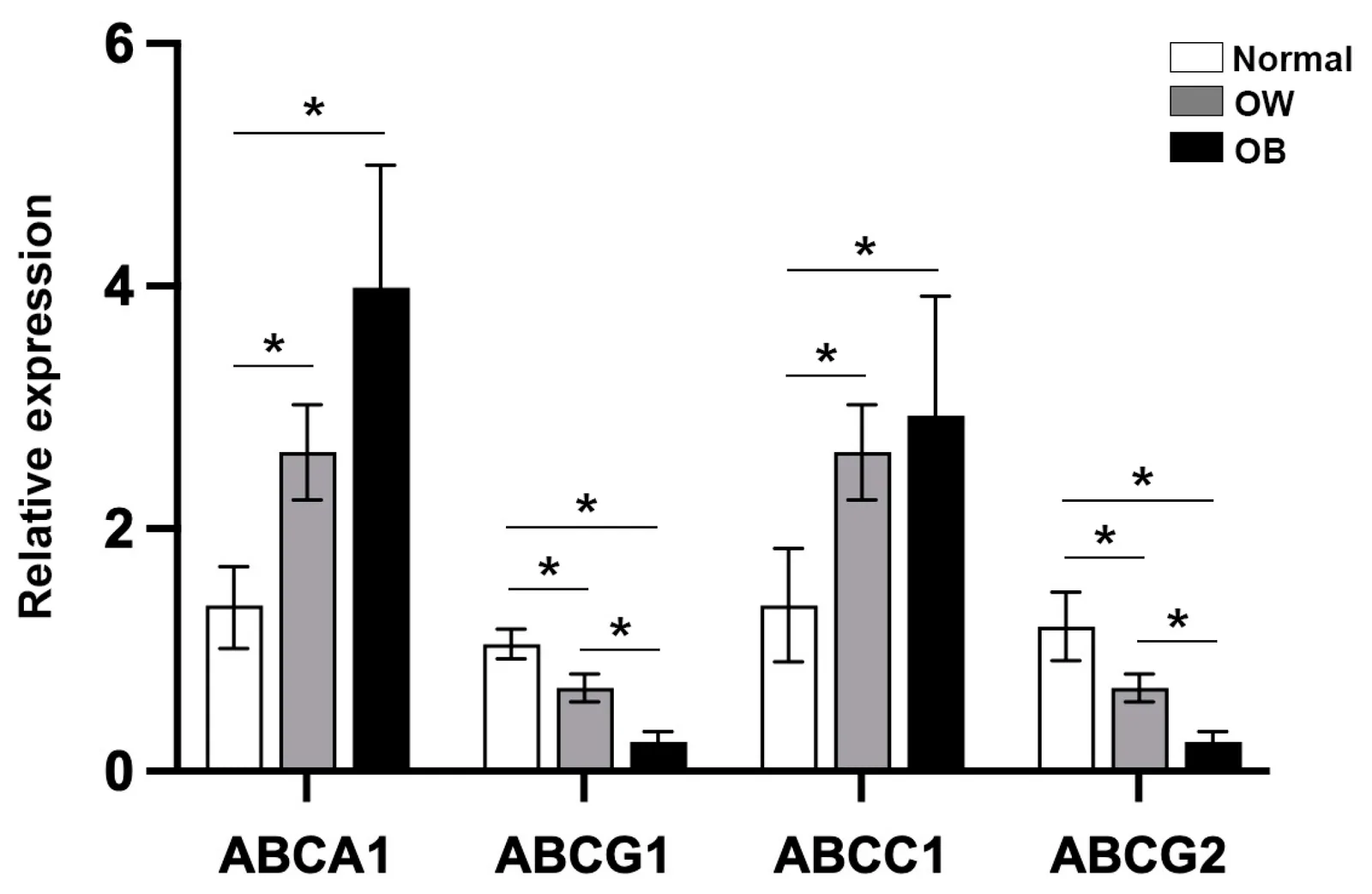
Expression of ABC transporters in newborns. Gene expression analysis of *ABCA1*, *ABCG1*, *ABCC1* and *ABCG2* in newborns of women classified according to their pregestational weight: normal (Normal, white), overweight (OW, gray) and obesity (OB, black). Statistical significance was assessed using the Kruskal–Wallis test, followed by Bonferroni-adjusted pairwise comparisons. Data are presented as mean ± SD. * *p* < 0.05.

**Table 1:** Maternal anthropometric and biochemical measurements according to the pregestational BMI category.

	Pregestational BMI Category [Kg/m^2^]		
	Normal 18.5 to <25.0 (*n* = 34)	Overweight 25.0 to <30.0 (*n* = 35)	Obese ≥30 (*n* = 23)
Age (years)	31.08 ± 5.51	32.60 ± 5.57	28.40 ± 4.06 ^c^
Pregestational BMI [Kg/m^2^]	23.12 ± 1.62	27.10 ± 1.60 ^a^	33.01 ± 2.64 ^b,c^
Gestational weight gain [Kg]	13.09 ± 4.42	9.59 ± 6.97	7.47 ± 5.25 ^b^
IWG [N (%)]	14 (41.20)	7 (20.00)	2 (8.70)
AWG [N (%)]	16 (47.10)	15 (42.90)	16 (69.60)
EWG [N (%)]	4 (11.80)	13 (37.10)	5 (21.70)
Primigesta [%]	21.40	10.50	20.00
Tobacco Smoking Before Pregnancy [N (%)]	13 (38.23)	9 (25.71)	9 (39.13)
Fasting glucose [mg/dL]	76.56 ± 10.80	77.76 ± 12.35	81.21 ± 12.67
Total Cholesterol [mg/dL]	209.32 ± 55.47	235.28 ± 36.29	233.18 ± 38.99
Triglycerides [mg/dL]	227.82 ± 71.39	244.12 ± 74.92	250.07 ± 91.85
HDL-C [mg/dL]	65.35 ± 10.47	56.70 ± 12.31	56.22 ± 10.19
LDL-C [mg/dL]	132.06 ± 24.10	145.12 ± 30.05	150.75 ± 30.12
TyG Index	9.07 ± 0.28	9.13 ± 0.39	9.09 ± 0.41
TyG-BMI	198.11 ± 16.29	248.65 ± 17.80 ^a^	298.82 ± 35.66 ^b,c^

BMI: body mass index; IWG: insufficient weight gain; AWG: adequate weight gain; EWG: excessive weight gain; HDL-C: high-density lipoprotein cholesterol; LDL-C: low-density lipoprotein cholesterol. Values represent mean ± SD. ^a^ *p* < 0.05 overweight versus normal; ^b^ *p* < 0.05 obese versus normal; ^c^ *p* < 0.05 obese versus overweight.

**Table 2:** Anthropometric and fetal biochemical parameters of newborns at birth according to maternal pregestational BMI category.

	Pregestational BMI Category [Kg/m^2^]		
	Normal 18.5 to <25.0 *n* = 25	Overweight 25.0 to <30.0 *n* = 27	Obese ≥30 *n* = 20
Gestational age at birth (weeks)	39.1 ± 1.07	38.63 ± 1.28	38.85 ± 1.66
Newborn weight [g]	3033.62 ± 373.83	3248.42 ± 520.52	3114.23 ± 550.97
Newborn sex			
Female [N (%)]	14 (56)	11 (40.7)	12 (60.0)
Male [N (%)]	11 (44)	16 (59.3)	8 (40.0)
Fasting glucose [mg/dL]	65.42 ± 8.21	62.95 ± 6.97	67.50 ± 6.68
Total cholesterol [mg/dL]	72.08 ± 14.58	71.23 ± 15.02	71.06 ± 17.60
Triglycerides [mg/dL]	40.94 ± 23.83	36.73 ± 9.50	38.11 ± 6.34
HDL-C [mg/dL]	31.36 ± 7.33	30.43 ± 7.94	29.20 ± 11.32
LDL-C [mg/dL]	40.72 ± 10.33	40.17 ± 13.64	39.45 ± 11.79
TyG Index	7.11 ± 1.79	7.01 ± 1.10	7.14 ± 0.54
FMF percentile	38.21 ± 25.88	43.26 ± 30.59	32.10 ± 29.56
FMF z score	−0.36 ± 0.82	−0.31 ± 1.19	−0.46 ± 1.14
FMB Percentile	47.50 ± 27.73	49.68 ± 31.56	38.30 ± 32.00
Intergrowth percentile	48.09 ± 23.96	41.77 ± 26.92	66.35 ± 29.81
Intergrowth z score	−0.86 ± 0.98	−0.25 ± 0.84	0.64 ± 1.13
SGA [N (%)]	5 (20)	4 (14.81)	6 (30)
AGA [N (%)]	18 (72)	17 (62.96)	12 (60)
LGA [N (%)]	2 (8)	6 (22.22)	2 (10)

Values represent mean ± SD. BMI: body mass index; HDL-C: high-density lipoprotein cholesterol; LDL-C: low-density lipoprotein cholesterol; FMF percentile: Fetal Medicine Foundation percentile; FMB percentile: Fetal Medicine Barcelona percentile; SGA: small for gestational age; AGA: adequate for gestational age; LGA: large for gestational age.

**Table 3:** Spearman correlation coefficients among maternal and newborn ABC transporter mRNA expression levels.

		Spearman Correlation Coefficients	*p*
***ABCA1*** **-Maternal**	*ABCG1*-Maternal	0.447	0.0001
	*ABCA1*-Newborn	0.623	0.017
***ABCA1*** **-Newborn**	*ABCG1*-Newborn	−0.526	0.0001
	*ABCC1*-Newborn	0.558	0.0001
	*ABCG2*-Newborn	−0.478	0.001
***ABCG1*** **-Newborn**	*ABCC1*-Newborn	−0.546	0.0001
	*ABCG2*-Newborn	0.748	0.0001
***ABCC1*** **-Newborn**	*ABCG2*-Newborn	−0.663	0.0001

**Table 4:** Spearman correlation analysis between ABC transporters expression, biochemical and anthropometrical parameters.

		Spearman Correlation Coefficients	*p*
***ABCA1*** **-Maternal**	Maternal fasting glucose	−0.508	0.037
***ABCG1*** **-Maternal**	FMF percentile	0.569	0.001
***ABCG2*** **-Maternal**	Maternal total cholesterol	−0.515	0.034
	FMF percentile	0.459	0.001
***ABCG1*** **-Newborn**	TyG-BMI Maternal	−0.752	0.001
***ABCC1*** **-Newborn**	TyG-BMI Maternal	0.605	0.001
***ABCG2*** **-Newborn**	TyG-BMI Maternal	−0.763	0.001

FMF percentile: Fetal Medicine Foundation percentile.

## Data Availability

The data sets used and analyzed during the current study are available from the corresponding author on reasonable request.

## Supplementary Materials

The following supporting information can be downloaded at https://www.mdpi.com/article/10.3390/metabo16100734/s1, Table S1. Adjusted Spearman correlation coefficients between maternal and newborn ABC mRNA expression levels after adjustment for potential confounding variables. Table S2. Adjusted Spearman correlation coefficients between maternal ABC mRNA expression, biochemical and anthropometrical parameters after adjustment for potential confounding variables. Table S3. Adjusted Spearman correlation coefficients for associations between neonatal ABC mRNA expression and biochemical and anthropometric parameters, controlling for potential confounding variables. Figure S1. Spearman correlations between ABC mRNA expression levels. Scatter plots illustrate the correlations between (A) maternal ABCA1 and maternal ABCG1 mRNA expression (*n* = 62) and (B) maternal and neonatal ABCA1 mRNA expression (*n* = 62). These associations remained statistically significant after adjustment for potential confounding variables, including maternal age, parity, smoking before pregnancy, and newborn sex. Figure S2. Spearman correlations between ABC transporter mRNA expression levels and biochemical and anthropometric parameters. Scatter plots illustrate the correlations between (A) maternal fasting glucose and maternal ABCA1 mRNA expression (*n* = 62), (B) maternal total cholesterol and maternal ABCG2 mRNA expression (*n* = 63), (C) maternal ABCG1 mRNA expression and Fetal Medicine Foundation Percentile (*n* = 56), and (D) maternal ABCG2 mRNA expression and Fetal Medicine Foundation Percentile (*n* = 63). These associations remained statistically significant after adjustment for potential confounding variables, including maternal age, parity, smoking before pregnancy, and newborn sex.

## Abbreviations

The following abbreviations are used in this manuscript:

ABC	ATP-binding cassette
AGA	Adequate for Gestational Age
AWG	Adequate weight gain
BMI	Body Mass Index
cDNA	Complementary Deoxyribonucleic acid
CTs	Cycles of threshold
EWG	Excessive weight gain
FMB	Fetal Medicine Barcelona
FMF	Fetal Medicine Foundation
GWG	Gestational weight gain
HDL-C	High-density lipoprotein cholesterol
IOM	Institute of Medicine
IWG	Insufficient weight gain
LDL-C	Low-density lipoprotein cholesterol
LGA	Large for Gestational Age
mRNA	Messenger Ribonucleic acid
PBMCs	Peripheral Blood Mononuclear Cells
SGA	Small for Gestational Age
TC	Total cholesterol
TG	Triglycerides
TyG Index	Triglyceride–glucose Index
TyG-BMI	Triglyceride–glucose–body mass Index
