# Supplementary material for: Pregestational Body Mass Index Is Associated with Differential Expression of ABC Transporter in the Maternal–Neonatal Dyad
Source: Metabolites. 2026 Sep 30;16(10):734. doi: 10.3390/metabo16100734 (PMC13707346; doi:10.3390/metabo16100734)
Supplement: Supplementary file 1 [file metabolites-16-00734-s001.zip › metabolites-4454763-supplementary.pdf]

Supplementary Materials

Table S1. Adjusted Spearman correlation coefficients between maternal and newborn ABC mRNA expression levels after adjustment for potential confounding variables

		**Age (years)**				**Parity**				**Smoking before Pregnancy**				**Newborn sex**			
		**< 35**		**≥ 35**		**Primigesta**		**Multiparous**		***No***		***Yes***		***Female***		***Male***	
		SCC	***p***	***SCC***	***p***	***SCC***	***p***	***SCC***	***p***	***SCC***	***p***	***SCC***	***p***	***SCC***	***p***	***SCC***	***p***
***ABCA1* Maternal**	ABCG1 Maternal																
	ABCC1 Maternal	.525	.001					.411	.001			.651	<0.0001	.381	.034	.534	.003
	ABCG2 Maternal			.525	.004												
	ABCA1 Newborn																
	ABCG1 Newborn			-.530	.004			-.422	.002			-.457	.032				
	ABCG2 Newborn			-.540	.014							-.541	.005				
***ABCA1* Newborn**	ABCG1 Newborn	-.684	<0.0001					-.531	<0.0001	-.468	.016			-.624	.002		
	ABCC1 Newborn	.570	.001	.530	.014			.568	<0.0001	.462	.015	.688	<0.0001			.675	.001
	ABCG2 Newborn	-.616	<0.0001					-.510	<0.0001	-.450	.036	-.452	.030	-.492	.020	-.601	.006
***ABCG1* Newborn**	ABCC1 Newborn	-.660	<0.0001					-.535	<0.0001	-.685	<0.0001	-.485	.030	-.639	.003	-.745	.001
	ABCG2 Newborn	.672	<0.0001	.792	<0.0001			.764	<0.0001	.479	.028	.906	<0.0001	.894	<0.0001	.713	.001
***ABCC1* Newborn**	ABCG2 Newborn	-.746	<0.0001					-.661	<0.0001	-.734	.000	-.623	.002	-.605	.004	-.674	.003

SCC: Spearman correlation coefficients

Table S2. Adjusted Spearman correlation coefficients between maternal ABC mRNA expression, biochemical and anthropometrical parameters after adjustment for potential confounding variables.

		Age (Years)				Parity				Smoking Before Pregnancy				Newborn Sex			
		<35		≥35		Primigesta		Multiparous		No		Yes		Female		Male	
		SCC	***p***	***SCC***	***p***	***SCC***	***p***	***SCC***	***p***	***SCC***	***p***	***SCC***	***p***	***SCC***	***p***	***SCC***	***p***
ABCA1 **Maternal**	Maternal Fasting Glucose																
	HDL-C Maternal									.296	.046	-.548	.003				
	TyG-IBM Index Maternal			.345	.049			.238	.041			.474	.009	.317	.047		
	TyG-IBM Newborn					-.761	0.017										
ABCG1 **Maternal**	FMF percentile																
	Fasting Glucose Maternal			.403	.033							.390	.045				
	HDL-C Newborn			-.540	.003												
ABCC1 **Maternal**	Intergrowth z-score									-.365	.040						
	FMF percentile									-.352	.048						
	Fasting Glucose Newborn			-0.426	0.021												
	Fasting Glucose Maternal							-.290	.025								
	HDL-C Maternal			-.421	.026							-.427	.030				
	TyG-IBM Maternal	.489	.001	.549	.002			.475	<0.0001	.564	<0.0001	.452	.018	.631	<0.0001	.372	.043
ABCG2 **Maternal**	HDL-C Maternal													.537	.002		
	Maternal Cholesterol																
	HDL-C Newborn									.392	.012			.366	.030		
	TyG Index Maternal	.364	.015					.321	.010	.489	.001						

SCC: Spearman correlation coefficients

Table S3. Adjusted Spearman correlation coefficients for associations between neonatal ABC mRNA expression and biochemical and anthropometric parameters, controlling for potential confounding variables.

		Age (Years)				Parity				Smoking				Product sex			
		<35		>35		Primigesta		Multiparous		No		Yes		Female		Male	
		**SCC**	**p**	**SCC**	**p**	**SCC**	**p**	**SCC**	**p**	**SCC**	**p**	**SCC**	**p**	**SCC**	**p**	**SCC**	**p**
***ABCA1* Newborn**	HDL-C Newborn	-.363	.029					-.405	.002					-.482	.009		
	TyG Index Maternal	.665	<0.0001														
	TyG-IBM Index Maternal							.589	<0.0001	.639	<0.0001	.425	.038	.523	.004	.575	.003
***ABCG1* Newborn**	Fasting Glucose Newborn							-.288	.041	.392	.012			-.527	.008		
	Fasting Glucose Maternal			-.409	.034	-.975	.005										
	TyG Index Maternal			-.507	.007												
	TyG-IBM Maternal	-.631	<0.0001	-.762	<0.0001			-.762	0.000	-.669	<0.0001	-.713	<0.0001	-.783	<0.0001	-.730	<0.0001
	TyG Index Newborn			-.398	.044												
***ABCC1* Newborn**	Fasting Glucose Newborn															-.422	.045
	HDL-C Maternal											-.513	.012				
	HDL-C Newborn													-.514	.009		
	TyG-IBM Index Maternal	.757	<0.0001					.598	<0.0001	.571	.001	.633	.001	.694	<0.0001	.659	.001
***ABCG2* Newborn**	HDL-C Maternal											.459	.024	.457	.029		
	HDL-C Newborn													.487	.014		
	TyG Index Maternal		-.498	.025								-.452	.023				
	TyG-IBM Index Maternal	-.747	<0.0001	-.776	<0.0001			-.773	<0.0001	-.737	<0.0001	-.765	<0.0001	-.796	<0.0001	-.753	<0.0001

SCC: Spearman correlation coefficients

Supplementary Figures

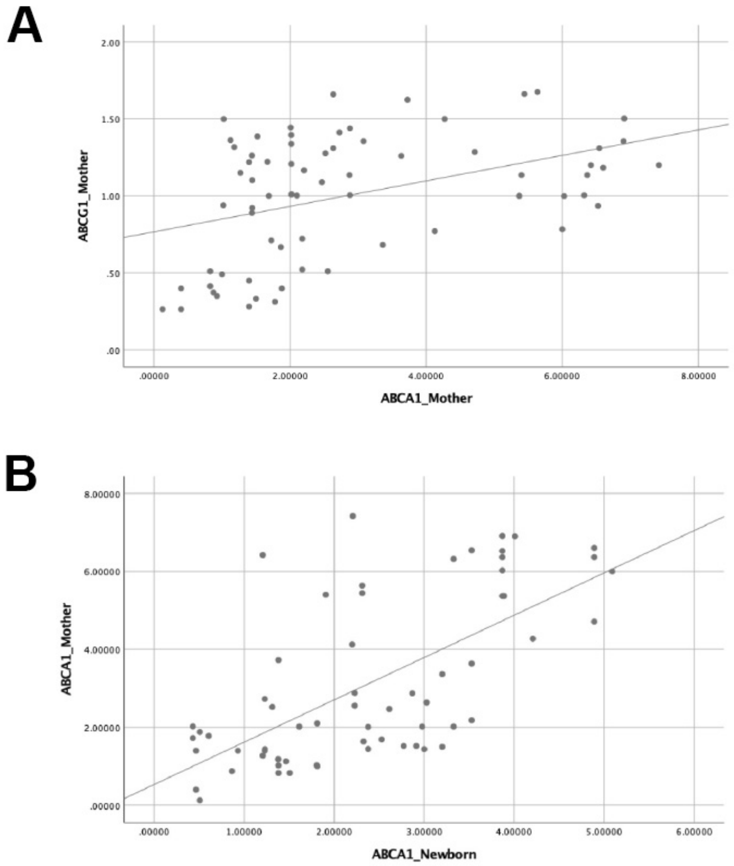

Figure S1. Spearman correlations between ABC mRNA expression levels. Scatter plots illustrate the correlations between (**A**) maternal *ABCA1* and maternal *ABCG1* mRNA expression (n = 62) and (**B**) maternal and neonatal *ABCA1* mRNA expression (n = 62). These associations remained statistically significant after adjustment for potential confounding variables, including maternal age, parity, smoking before pregnancy, and newborn sex.

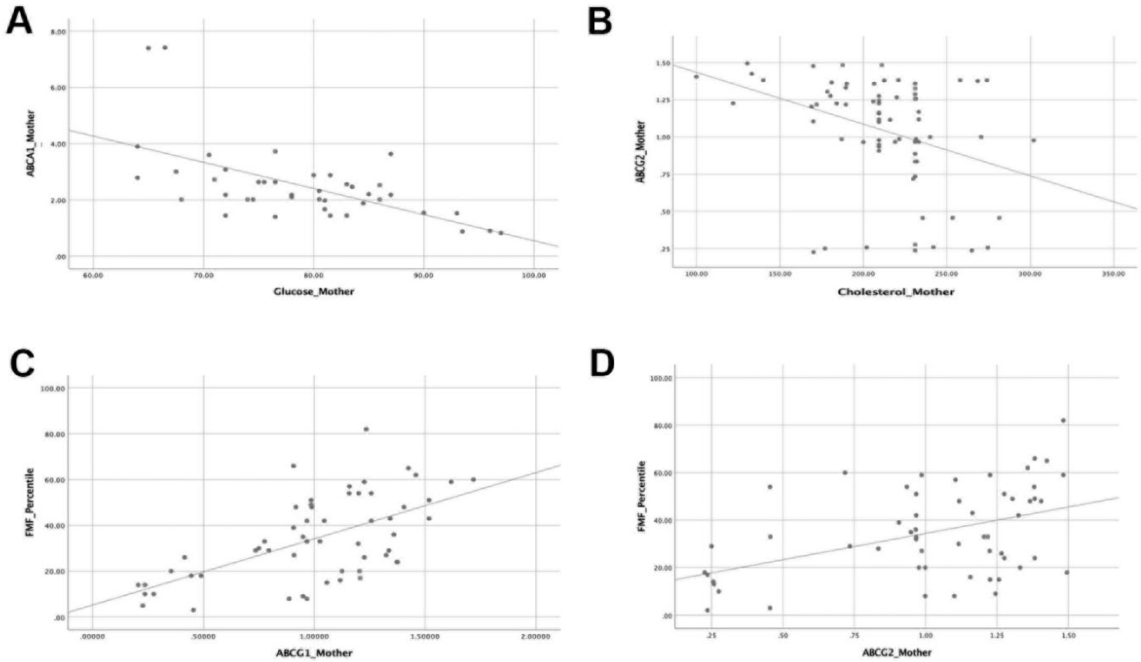

Figure S2. Spearman correlations between ABC transporter mRNA expression levels and biochemical and anthropometric parameters. Scatter plots illustrate the correlations between (**A**) maternal fasting glucose and maternal *ABCA1* mRNA expression (n = 62), (**B**) maternal total cholesterol and maternal *ABCG2* mRNA expression (n = 63), (**C**) maternal *ABCG1* mRNA expression and Fetal Medicine Foundation Percentile (n = 56), and (**D**) maternal *ABCG2* mRNA expression and Fetal Medicine Foundation Percentile (n = 63). These associations remained statistically significant after adjustment for potential confounding variables, including maternal age, parity, smoking before pregnancy, and newborn sex.
